# Supplementary material for: The causal role of circulating inflammatory markers in osteoporosis: a bidirectional Mendelian randomized study
Source: Front Immunol. 2024 Jul 18;15:1412298. doi: 10.3389/fimmu.2024.1412298 (PMC11291241; doi:10.3389/fimmu.2024.1412298)
Supplement: Supplementary file 1 [file DataSheet_1.zip › supplementary material/OSTEOPOROSIS-CIM/GCST90274805.tsv.gz_leave_one_out.pdf]

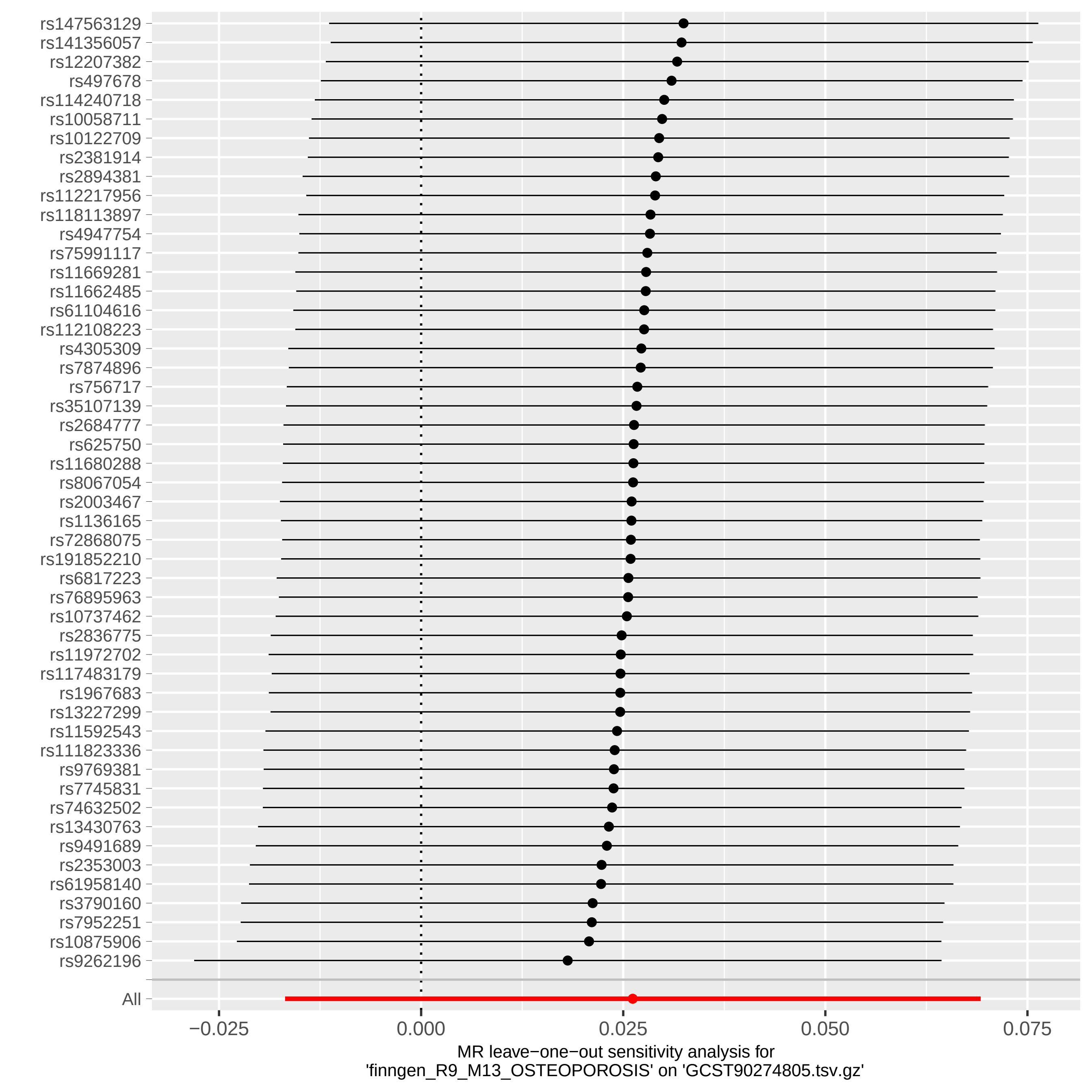
rs147563129
rs141356057
rs12207382
rs497678
rs114240718
rs10058711
rs10122709
rs2381914
rs2894381
rs112217956
rs118113897
rs4947754
rs75991117
rs11669281
rs11662485
rs61104616
rs112108223
rs4305309
rs7874896
rs756717
rs35107139
rs2684777
rs625750
rs11680288
rs8067054
rs2003467
rs1136165
rs72868075
rs191852210
rs6817223
rs76895963
rs10737462
rs2836775
rs11972702
rs117483179
rs1967683
rs13227299
rs11592543
rs111823336
rs9769381
rs7745831
rs74632502
rs13430763
rs9491689
rs2353003
rs61958140
rs3790160
rs7952251
rs10875906
rs9262196
All
−0.025
0.000
0.025
0.050
0.075
MR leave−one−out sensitivity analysis for
'finngen_R9_M13_OSTEOPOROSIS' on 'GCST90274805.tsv.gz'
